# Control Design for Signal Transduction Networks

**DOI:** 10.4137/bbi.s2116

**Published:** 2009-02-09

**Authors:** Chun-Liang Lin, Yuan-Wei Liu, Chia-Hua Chuang

**Affiliations:** Department of Electrical Engineering, National Chung Hsing University, Taichung 402, Taiwan, ROC

**Keywords:** signal transduction networks, biochemical networks, systems biology, control, stability

## Abstract

Signal transduction networks of biological systems are highly complex. How to mathematically describe a signal transduction network by systematic approaches to further develop an appropriate and effective control strategy is attractive to control engineers. In this paper, the synergism and saturation system (S-systems) representations are used to describe signal transduction networks and a control design idea is presented. For constructing mathematical models, a cascaded analysis model is first proposed. Dynamic analysis and controller design are simulated and verified.

## Introduction

Recently, interdisciplinary studies are becoming popular; one of the most attractive studies is to combine both biological systems and control engineering.^1^,^2^,^3^ Signal transduction networks of biological systems are characterized as high complexity because they are composed of many biochemical reactions. Typical modeling process for that kind of systems may involve the following issues: modeling, dynamic analysis and steady-state analysis.^4^,^5^ However, the complexity of cellular signal transduction network is in general incomprehensible. Thus, an effective method to develop a mathematically equivalent model of the biochemical networks is highly desirable.

The synergism and saturation system (S-system)^6^ has been a well-studied approach in modeling biochemical networks which characterizes the signal transduction networks. It was shown that the S-system representation in terms of ordinary differential equations (ODEs) is capable of capturing the behavior of biochemical dynamics. Applying logarithm on the state variables linearizes the state equations of the S-system at steady state. Based on the linearized S-system, it is possible to analyze and predict the S-system behavior rather than directly resorting to the original nonlinear model. In,^1^ a linearized S-system was derived and the robust stability analysis was conducted. In^7^, the properties of cascaded signal transduction pathway, robust and optimal design of system circuit and rule of gene regulation were introduced. Based on the steady-state analyses of the S-system model, a robust control method is proposed for biochemical networks via feedback and feedforward biochemical circuits was proposed by.^8^,^9^ The proposed robust circuit design schemes provide a systematic method with applications in synthetic circuit design for biotechnological purpose.

The purpose of this paper is to model and analyze the signal transduction networks in biological systems and transform the mathematical model from the uncontrollable and nonlinear form to a controllable and linear form. We adopt the S-system and the Michaelis-Menten rate law to drive a mathematical model for signaling transduction networks. For simplifying the construction of the mathematical model, a method called ‘cascaded analysis model’ is proposed. The model is intended to be used for constructing a simplified mathematical model in the form of S-systems. This method avoids directly solving the entire mathematical model, which could be extremely complicated in structure. Rather, the problem can be broken down into smaller partitions to lessen computational burden.

Stability analysis is also presented. The Lyapunov stability theory is applied to determine stability of the S-system under Taylor expansion which could be used to estimate stability margin of the biological system without control. A new control design idea for the nonlinear S-systems is then attempted. By applying feedback linearization,^10^ a proper coordinate transformation can be derived to establish an effective method for control of the nonlinear systems with smooth nonlinearities, which are nonlinear in their state variables but linear in their control variables. For S-systems, dependent variables can be chosen as state variables and independent variables as control inputs. On the basis of,^11^ one can convert the nonlinear system into a linearized controllable system. By choosing an appropriate controller, it’s shown that the baseline steady state can be driven to the desired level in a given period.

## Methods

### Modeling of signal transduction networks

A signal transduction network includes many scaffolds which can be bound with molecules. Since the entire pathways which would influence reactions are, in general, too large to be conducted, an effective method for constructing a simplified mathematical model is highly desirable. To this aim, a method is attempted here to construct a simplified model.

First, consider the scaffold protein with *n* binding domains and each domain can be bound with one molecule. Also, define all states and pathways of the scaffold protein as follows

(1) $$S=\sum_{i=0}^{n}\left(\begin{array}{c} n\\ i \end{array}\right)$$

(2) $$P=\sum_{i=0}^{n-i}\left(\begin{array}{c} n\\ i \end{array}\right)\left(\sum_{j=1}^{n-i}2^{j-1}\right)$$

with

(3) $$\left(\begin{array}{c} n\\ i \end{array}\right)=\frac{n!}{i!(n-i)!}$$

where *S*, *P*, and *n* denote, respectively, states, pathways and binding domains.

Second, consider the case where scaffold protein can combine with one molecule each time. Under this situation, one can neglect the redundant pathways. To simplify the overly complicated structure, the new pathways can be written as follows

(4) $$P=\sum_{i=0}^{n}\left(\begin{array}{c} n\\ i \end{array}\right)(n-i)$$

Third, define states and molecules as the state variables *x* and reject the reactions on the pathways. Adopting the Michaelis-Menten rate law,^12^ each reaction can be represented as an ODE. Consequently, the general equations which describe the temporal changes in the biochemical system^13^ can be formulated as

(5) $$\begin{array}{cc} {\dot{x}}_{i}=V_{i}^{+}-V_{i}^{-}, & i=1,2,\ldots ,n\\ V_{i}^{+}=\alpha_{i}\prod_{j=1}^{n+m}x_{j}^{g_{ij}}, & V_{i}^{-}=\beta_{i}\prod_{j=1}^{n+m}x_{j}^{h_{ij}} \end{array}$$

where $V_{i}^{+}=V_{i}^{+}(x_{1},\;x_{2},\ldots ,x_{n},\;x_{n+1},\ldots ,x_{n+m})$ and $V_{i}^{-}=V_{i}^{-}(x_{1},\;x_{2},\ldots ,x_{n},\;x_{n+1},\ldots ,x_{n+m})$ are general functions of the dependent variables *x*_1_, *x*_2_, ..., *x_n_* and independent variables *x_n_* _+ 1_, *x_n_* _+ 2_, ..., *x_n_* _+_ *_m_* *α_i_* and *β_i_* are rate constants; *g_ij_* and *h_ij_* are the kinetic orders.

Considering the steady state of the system, all rate constants and variables in (5) are given as nonzero and take the logarithm in (5). Defining *y_i_* = ln*x_i_* and arranging all terms for *y_i_* to one side and other terms to another side gives

(6) $$\text{ln}\left(\frac{\beta_{i}}{\alpha_{i}}\right)=\sum_{j=1}^{n+m}g_{ij}y_{j}-\sum_{j=1}^{n+m}h_{ij}y_{j},\;\;i=1,2,\ldots ,n$$

Next, define $b_{i}=\text{ln}\left(\frac{\beta_{i}}{\alpha_{i}}\right)$ and *a_ij_* = *g_ij_* − *h_ij_*.

A general S-system with *n* dependent variables and *m* independent variables can then be characterized in the matrix form as

(7) $$A_{D}{\vec{y}}_{D}=\vec{b}-A_{I}{\vec{y}}_{I}$$

where

$$\begin{array}{l} A_{D}=\left[\begin{array}{cccc} a_{11} & a_{12} & \cdots & a_{1n}\\ a_{21} & a_{22} & \cdots & a_{2n}\\ \vdots & \vdots & \ddots & \vdots \\ a_{n_{1}} & a_{n_{2}} & \cdots & a_{n_{n}} \end{array}\right],{\vec{y}}_{D}=\left[\begin{array}{c} y_{1}\\ y_{2}\\ \vdots \\ y_{n} \end{array}\right],\vec{b}=\left[\begin{array}{c} b_{1}\\ b_{2}\\ \vdots \\ b_{n} \end{array}\right],\\ A_{I}=\left[\begin{array}{cccc} a_{1,n+1} & a_{1,n+2} & \cdots & a_{1,n+m}\\ a_{2,n+1} & a_{2,n+2} & \cdots & a_{2,n+m}\\ \vdots & \vdots & \ddots & \vdots \\ a_{n,n+1} & a_{n,n+2} & \cdots & a_{n,n+m} \end{array}\right],{\vec{y}}_{I}=\left[\begin{array}{c} y_{n+1}\\ y_{n+2}\\ \vdots \\ y_{n+m} \end{array}\right] \end{array}$$

with the subscripts *D* and *I* meant dependent and independent variables respectively.

According to (7), one can obtain the steady states *y_i_*, *i* = 1, ..., *n* given by

(8) $${\vec{y}}_{D}=A_{D}^{-1}(\vec{b}-A_{I}{\vec{y}}_{I})$$

using the pre-described procedures, the originally complicated system could be transformation into an analyzable form.

The Michaelis-Menten equation for biological systems has been applied to investigate the concentration change of metabolites in each pathway of biochemical networks, and the concentration change equations are further expressed as ordinary differential equations. However, it may cost significant computation time to analyze all cellular signal reactions and interactions which are not all important or critical to the signal transduction networks. How to remove the redundant parts is an issue.

**Figure 1.**
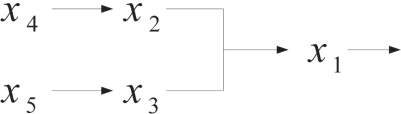
Basic reaction in biological system.

**Figure 2.**
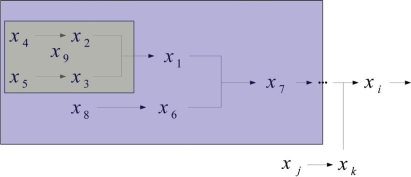
Complete cascaded analysis model; the small block is the 1st cascaded layer; the large block denotes the 2nd cascaded layer.

To simplify the analysis, we propose a cascaded analysis model to analyze the system with a simpler structure. A molecule that combined with a scaffold protein is a basic reaction in the mathematical model. This reaction can be described, for example, as a signal transduction pathway in Figure 1. After estimating all parameters of the S-system, one can compute the output concentration *x*_1_ at the steady state. One then cascades the output concentration with a new molecule to generate a new signal transduction pathway. For the same reason, one can cascade molecules to construct a complete mathematical model as shown in Figure 2. Repeating the steps, one can construct a mathematical model which is easier than constructing the model at a time.

**Figure 3.**
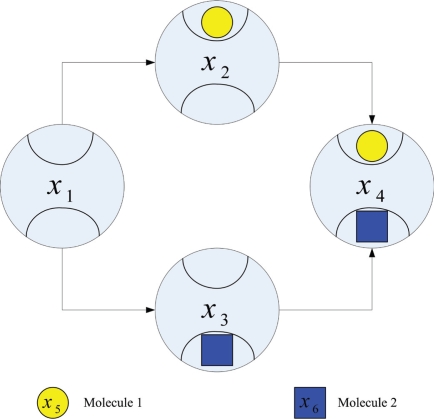
Reduced model with two binding domains.

**Figure 4.**
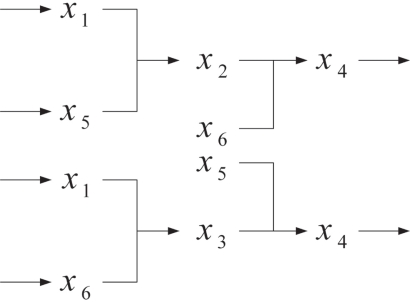
Constructing the signal transduction network by signal transduction pathways.

The method is demonstrated by a signal transduction network model with one scaffold protein and two binding domains in Figure 3. From (1) and (2), the number of states and pathways are 4 and 5, respectively. We neglect the redundant pathways described by (4) to simplify the complete model. The number of pathways of the new model becomes 4. We define states (*S*) and molecules as state variables *x_i_* and implement the pathways to reactions. We can then modify reactions and construct new signal transduction pathways as shown in Figure 4.

**Figure 5.**
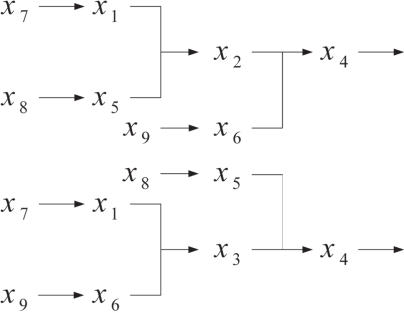
Adding independent variables to modify the signal trans-duction network

**Figure 6.**
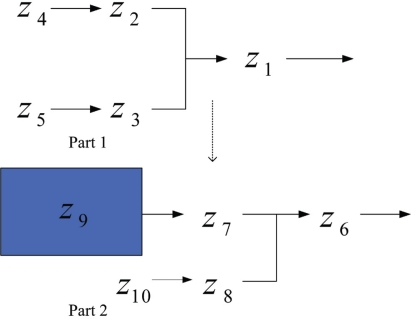
Example of cascaded analysis model.

On the basis of the signal transduction pathways in Figure 4, three independent variables (*x*_7_, *x*_8_, *x*_9_), which are outside signal of the signal transduction networks, are introduced to construct the analyzable model (Fig. 5). Applying the cascaded analysis model and considering the top part in Figure 5, we separate the pathways into two parts and define a new variable *z_i_* to substitute *x_i_* as indicated in Figure 6.

Consider part 1 in Figure 6, the system includes three dependent variables (*z*_1_, *z*_2_, *z*_3_) and two independent variables (*z*_4_, *z*_5_), and the fluxes contain variables (*V* *^+^*, *V*^−^). The S-system is built as

(9) $$\begin{array}{l} {\dot{z}}_{1}=\alpha_{1}z_{2}^{g_{12}}z_{3}^{g_{13}}-\beta_{1}z_{1}^{h_{11}},\\ {\dot{z}}_{2}=\alpha_{2}z_{4}^{g_{24}}-\beta_{2}z_{2}^{h_{22}}z_{3}^{h_{23}},\\ {\dot{z}}_{3}=\alpha_{3}z_{5}^{g_{35}}-\beta_{3}z_{2}^{h_{32}}z_{3}^{h_{33}},\\ z_{4},z_{5}=\text{constant} \end{array}$$

From which one can get the steady states of all state variables by taking logarithm on (9):

(10) $$\begin{array}{l} g_{12}\text{ln}z_{2}+g_{13}\text{ln}z_{3}=\text{ln}\left(\overset{\beta_{1}}{\;}/\underset{\alpha_{1}}{\;}\right)+h_{11}\text{ln}\;z_{1},\\ g_{24}\text{ln}z_{4}=\text{ln}\left(\overset{\beta_{2}}{\;}/\underset{\alpha_{2}}{\;}\right)+h_{22}\text{ln}\;z_{2}+h_{23}\text{ln}\;z_{3},\\ g_{35}\text{ln}z_{5}=\text{ln}\left(\overset{\beta_{3}}{\;}/\underset{\alpha_{3}}{\;}\right)+h_{32}\text{ln}\;z_{2}+h_{33}\text{ln}\;z_{3} \end{array}$$

Using the notations defined in (7), one can determine the steady state ${\vec{y}}_{D}$ by solving

(11) [a11a12a130a22a230a32a33][y1y2y3]=[b1b2−a24y4b3−a35y5]

Similarly, the S-system model for part 2 in Figure. 6 can be constructed as follows

(12) $$\begin{array}{l} {\dot{z}}_{6}=\alpha_{6}z_{7}^{g_{67}}z_{8}^{g_{68}}-\beta_{6}z_{6}^{h_{66}},\\ {\dot{z}}_{7}=\alpha_{7}z_{9}^{g_{79}}-\beta_{7}z_{7}^{h_{77}}z_{8}^{h_{78}},\\ {\dot{z}}_{8}=\alpha_{8}z_{10}^{g_{8,10}}-\beta_{8}z_{7}^{h_{87}}z_{8}^{h_{88}},\\ z_{9},z_{10}=\text{constant} \end{array}$$

The response time of each stage in the cascaded analysis model is governed by the degradation rate *α_i_* and *β_i_* of the protein at the stage of the cascaded analysis model. Using the cascaded analysis model, the original model can be replaced by a simplified one, which would be useful while constructing the signal transduction networks for analysis purpose.

### Stability analysis

Most chemical reactions in biological systems operate at a steady-state level, and normal concentrations in biological systems are maintained by regulatory mechanisms that stabilize the steady states. Effective regulation makes the concentrations return to steady states after being effected by external stimulation.

In general, the power-law representation in biological systems can be considered as a canonical nonlinear system. By performing Taylor expansion, the power-law representation can be employed as a piecewise expression. It provides a global representation of which validity and accuracy can be governed.^14^ On the other hand, the power-law representation can be employed as a local representation. Its accuracy within a neighborhood can be justified by investigating the effect resulting from the residual dynamics.

Consider, for instance, an S-system with two variables given by

(13) $$\begin{array}{l} {\dot{x}}_{1}=F_{1}(x_{1},x_{2})=\alpha_{1}x_{2}^{g_{12}}-\;\beta_{1}x_{1}^{h_{11}},\\ {\dot{x}}_{2}=F_{2}(x_{1},x_{2})=\alpha_{2}x_{1}^{g_{21}}-\;\beta_{2}x_{2}^{h_{22}} \end{array}$$

By applying the second-order Taylor expansion around the operation point (*x*_10_, *x*_20_) gives

$$F_{1}(x_{1},x_{2})=a_{11}x_{1}+a_{12}x_{2}+b_{1}+(c_{1}x_{1}^{2}+d_{1}x_{2}^{2})$$

where

$$\begin{array}{l} a_{11}=h_{11}(h_{11}-2)\beta_{1}x_{10}^{h_{11}-1},\\ a_{12}=-g_{12}(g_{12}-2)\alpha_{1}x_{20}^{g_{12}-1},\\ b_{1}=F(x_{10},x_{20})+\frac{1}{2}[-h_{11}(h_{11}-3)\times \\ \;\;\;\;\;\;\beta_{1}x_{10}^{h_{11}}-g_{12}(g_{12}-3)\alpha_{1}x_{20}^{g_{12}}],\\ c_{1}=\frac{-h_{11}(h_{11}-1)\beta_{1}x_{10}^{h_{11}-2}}{2},\\ d_{1}=\frac{g_{12}(g_{12}-1)\alpha_{1}x_{20}^{g_{12}-2}}{2} \end{array}$$

Similarly,

$$F_{2}(x_{1},x_{2})=a_{21}x_{1}+a_{22}x_{2}+b_{2}+(c_{2}x_{1}^{2}+d_{2}x_{2}^{2})$$

where

$$\begin{array}{l} a_{21}=h_{22}(h_{22}-2)\beta_{2}x_{10}^{h_{22}-1},\\ a_{22}=-g_{21}(g_{21}-2)\alpha_{2}x_{20}^{g_{21}-1},\\ b_{2}=F_{2}(x_{10},x_{20})+\frac{1}{2}[-h_{22}(h_{22}-3)\\ \;\;\;\;\;\;\times \beta_{2}x_{10}^{h_{22}}-g_{21}(g_{21}-3)\alpha_{2}x_{20}^{g_{21}}],\\ c_{2}=\frac{-h_{22}(h_{22}-1)\beta_{2}x_{10}^{h_{22}-2}}{2},\\ d_{2}=\frac{g_{21}(g_{21}-1)\alpha_{2}x_{20}^{g_{21}-2}}{2} \end{array}$$

The linearized S-system becomes

(14) $$\dot{x}\approx Ax+\Delta A(x)+b$$

where

$$A=\left[\begin{array}{cc} a_{11} & a_{12}\\ a_{21} & a_{22} \end{array}\right],\;\;\;\Delta A(x)\left[\begin{array}{c} c_{1}x_{1}^{2}+d_{1}x_{2}^{2}\\ c_{2}x_{1}^{2}+d_{2}x_{2}^{2} \end{array}\right]$$

and *x* = [*x*_1_ *x*_2_]*^T^* and *b* = [*b*_1_ *b*_2_]*^T^*; Δ*A*(*x*) denotes the residual error.

Consider the stability of the linearized S-system, the residual error Δ *A*(*x*) satisfies

(15) $${\left\|\Delta A(x)\right\|}_{2}\leq \alpha {\left\|x\right\|}_{2},\;\;\;\;\Delta A(x)\in {\mathbb{R}}^{n}$$

where ‖·‖_2_ denotes the Euclidean norm. Given an arbitrarily chosen *Q* = *Q^T^* > 0 and *A* is stable, by the Lyapunov stability theory, there will exist a solution *P* = *P^T^* > 0 to the following matrix equality:

(16) $$A^{T}P+PA=-2Q$$

Using the Rayleigh principle it can be shown that the whole system of (14), without the bias term, would be asymptotically stable provided that:^15^

(17) $$\alpha <\frac{\lambda_{\text{min}}(Q)}{\lambda_{\text{max}}(P)}$$

It should be noted that Taylor expansion will not change stability of the system. The purpose here is to estimate stability margin of the biological system without control.

### Control design

Feedback linearization is popular for nonlinear control designs.^16^ As it was shown by^11^ that a nonlinear system can be casted into a linearized controllable system. On the basis of a proper coordinate transformation, feedback linearization establishes a convenient tool for the control design of the nonlinear systems. This form is applicable for the biochemical systems given as follows

(18) $$\dot{x}(t)=f(x)+l(x)u$$

where *x* ∈ ℝ*^n^* and *u* ∈ ℝ*^m^*.

One can formulate the feedback linearization problem as follows. Define the nonlinear feedback control law as:

(19) u=A(x)+B(x)v

where *A*(*x*) is an *m*-dimensional vector field, *B*(*x*) is an *m* × *m* matrix and *v* is an *m*-dimensional vector.

Define a coordinate mapping as

(20) ψ:z=ψ(x)

such that the affine nonlinear control system (18) is transformed into a linear controllable system. For instance, by considering the nominal system (18), there exists a coordinate transformation as follows

(21) $$\left[\begin{array}{c} z_{1}\\ z_{2}\\ \vdots \\ z_{n} \end{array}\right]=\left[\begin{array}{c} \psi_{1}(x)\\ \psi_{2}(x)\\ \vdots \\ \psi_{n}(x) \end{array}\right]=\left[\begin{array}{c} h(x)\\ L_{f}h(x)\\ \vdots \\ L_{f}^{n-1}h(x) \end{array}\right]$$

where the Lie derivative $L_{f}h(x)=\frac{\partial h(x)}{\partial x}f$ is the direction derivative of *h* along the direction of *f* and one can find *h*(*x*) satisfying

(22) $$\begin{array}{l} L_{1}L_{f}^{k-1}h(x)=0,\;\;\;\;k=1,2,\ldots ,n-1\\ L_{1}L_{f}^{n-1}h(x)\neq 0 \end{array}$$

so that (18) can be transformed into a linear form as follows

(23) $$\dot{z}=Az+Bv$$

where

$$A=\left[\begin{array}{ccccc} 0 & 1 & 0 & \cdots & 0\\ 0 & 0 & 1 & \cdots & 0\\ \vdots & \vdots & \cdots & \ddots & \vdots \\ 0 & 0 & 0 & \cdots & 1\\ 0 & 0 & 0 & \cdots & 0 \end{array}\right],\;\;B=\left[\begin{array}{c} 0\\ 0\\ \vdots \\ 0\\ 1 \end{array}\right]$$

and one can choose a control law as follows

(24) $$u=\frac{-L_{f}^{n}h(x)}{L_{l}L_{f}^{n-1}h(x)}+\frac{1}{L_{l}L_{f}^{n-1}h(x)}v$$

where we have used the following definition^17^

$$L_{l}L_{f}^{i-1}h(x)=\sum_{j=1}^{n}\frac{\partial L_{f}^{i-1}}{\partial x_{j}}l(x),\;\;i=1,2,\ldots ,n$$

and

$$\begin{array}{l} L_{f}^{0}h(x)=h(x),\\ L_{f}^{k}h(x)=L_{f}L_{f}^{k-1}h(x)=〈dL_{f}^{k-1},f(x)〉,\;\;k\geq 1 \end{array}$$

Next, consider the following generalized representation of the nonlinear system with uncertainties as follows

(25) $$\dot{x}=f(x)+\Delta f(x)+l(x)u$$

There exists smooth function Δ f^*^(*x*) in ℝ*^n^* such that the uncertainties in (18), for all *x* ∈ ℝ*^n^*, satisfy the matching condition:

(26) $$\Delta f(x)=l(x)\Delta f^{*}(x)$$

Substituting the matched uncertainties (26) into (18), by the feedback linearization, it can be transformed into

(27) $$\begin{array}{l} {\dot{z}}_{1}=z_{2}+(L_{l}h)\Delta f^{*}+(L_{l}h)u,\\ {\dot{z}}_{2}=z_{3}+(L_{l}L_{f}h)\Delta f^{*}+(L_{l}L_{f}h)u,\\ \vdots \\ {\dot{z}}_{n}=L_{f}^{n}h+(L_{l}L_{f}^{n-1}h)\Delta f^{*}+(L_{l}L_{f}^{n-1}h)u \end{array}$$

Substituting (22) and (24) into (27), one can finally transform (27) into the following form:

(28) $$\begin{array}{l} {\dot{z}}_{1}=z_{2},\\ {\dot{z}}_{2}=z_{3},\\ \vdots \\ {\dot{z}}_{n}=v+(L_{l}L_{f}^{n-1}h)\Delta f^{*} \end{array}$$

Assume that *v* is given by

(29) v=−Kz

where *K* is a constant row vector. Substituting (29) into (23) yields

(30) $$\dot{z}=A_{c}z+B\Delta B(z)$$

where

$$A_{c}=\left[\begin{array}{ccccc} 0 & 1 & 0 & \cdots & 0\\ 0 & 0 & 1 & \cdots & 0\\ \vdots & \vdots & \cdots & \ddots & \vdots \\ 0 & 0 & 0 & \cdots & 1\\ -k_{1} & -k_{2} & -k_{3} & \cdots & -k_{n} \end{array}\right],\;\;B=\left[\begin{array}{c} 0\\ 0\\ \vdots \\ 0\\ 1 \end{array}\right]$$

and $\Delta B=\left((L_{l}L_{f}^{n-1}h)\Delta f^{*}\right)\circ \psi^{-1}(z)$ where ∘ denotes the composition of functions. Thus the function $(L_{l}L_{f}^{n-1}h)\Delta f^{*}$ can be transformed from *x*-domain to *z*-domain.

In the first stage of control design, the gain vector *K* should be chosen so that *A_c_* would be stable. Stability analysis of the uncertain control system is next carried out by using Lyapunov stability theory.

To proceed, a Lyapunov candidate function is defined as follows

(31) $$V(z)=z^{T}Pz$$

where *P* = *P^T^* > 0.

Taking derivative with respect to time gives

(32) $$\dot{V}(z)=z^{T}(A_{c}^{T}P+PA_{c})z+2z^{T}PB\Delta B(z)$$

As *A_c_* has been a stable matrix, for any *Q* = *Q^T^* > 0, there is a unique symmetric positive definite solution *P* to the following Lyapunov matrix equation:

(33) $$A_{c}^{T}P+PA_{c}=-Q$$

Substituting (33) into (32) gives

(34) $$\dot{V}\leq -\lambda_{\text{min}}(Q){\left\|z\right\|}_{2}^{2}+2{\left\|z^{T}PB\Delta B(z)\right\|}_{2}$$

If there exists a constant *α* with λ_min_(*Q*) > *α* such that

(35) $$2{\left\|z^{T}PB\Delta B(z)\right\|}_{2}\leq \alpha {\left\|z\right\|}_{2}^{2}$$

Then (34) becomes

(36) $$\dot{V}\leq -\left[\lambda_{\text{min}}(Q)-\alpha \right]{\left\|z\right\|}_{2}^{2}$$

Clearly, one can have *V̇*(*x*) < 0, i.e. asymptotic stability of the uncertain system under feedback control, by the suitably chosen matrix *Q*.

## Demonstrative Example

### Cascaded analysis model

According to Figure 3 and (9), we set all parameters to construct the S-system as follows

(37) $$\begin{array}{l} {\dot{z}}_{1}=2z_{2}^{0.5}z_{3}^{0.5}-2z_{1}^{1},\\ {\dot{z}}_{2}=2z_{4}^{0.5}-2z_{2}^{0.6}z_{3}^{0.4},\\ {\dot{z}}_{3}=2z_{5}^{0.5}-2z_{2}^{0.4}z_{3}^{0.6},\\ z_{4}=0.5,\\ z_{5}=0.5 \end{array}$$

The dynamic analysis was simulated by using the program: Power Law Analysis and Simulation (PLAS). The output concentration of the S-systems is obtained as *z*_1_ = 0.701069 On the basis of the output concentration and (12), we have the parameters for the next stage model as follows

(38) $$\begin{array}{l} {\dot{z}}_{6}=2z_{7}^{0.5}z_{8}^{0.5}-2z_{6}^{1},\\ {\dot{z}}_{7}=1.68z_{9}^{0.5}-1.68z_{7}^{0.6}z_{8}^{0.4},\\ {\dot{z}}_{8}=2z_{10}^{0.5}-2z_{7}^{0.4}z_{8}^{0.6},\\ z_{9}=0.701,\\ z_{10}=0.5 \end{array}$$

The output concentration of the S-systems is *z*_6_ = 0.7696588.

**Figure 7.**
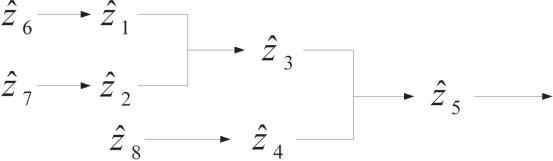
Reference signal transduction network for the complete model.

Consider the complete S-system with its signal transduction pathways illustrated as in Figure 7. The S-system can be represented as

(39) $$\begin{array}{l} {\dot{\hat{z}}}_{1}=2{\hat{z}}_{6}^{0.5}-2{\hat{z}}_{1}^{0.6}{\hat{z}}_{2}^{0.4},\\ {\dot{\hat{z}}}_{2}=2{\hat{z}}_{7}^{0.5}-2{\hat{z}}_{1}^{0.4}{\hat{z}}_{2}^{0.6},\\ {\dot{\hat{z}}}_{3}=2{\hat{z}}_{1}^{0.5}{\hat{z}}_{2}^{0.5}-2{\hat{z}}_{3}^{0.4}{\hat{z}}_{4}^{0.6},\\ {\dot{\hat{z}}}_{4}=2{\hat{z}}_{8}^{0.5}-2{\hat{z}}_{3}^{0.4}{\hat{z}}_{4}^{0.6},\\ {\dot{\hat{z}}}_{5}=2{\hat{z}}_{3}^{0.5}{\hat{z}}_{4}^{0.5}-2{\hat{z}}_{5}^{1},\\ {\dot{\hat{z}}}_{6}={\hat{z}}_{7}={\hat{z}}_{8}=1 \end{array}$$

**Figure 8.**
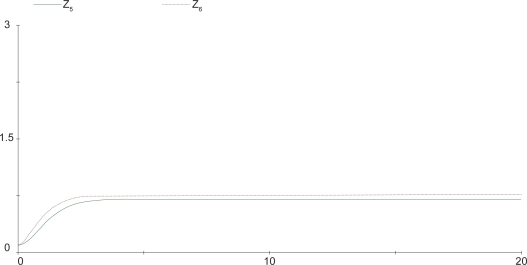
Comparison of the output concentration. Dashed line denotes the output concentration of the cascaded analysis model, solid line denotes the output concentration of the reference system.

The steady output concentration of the S-systems is *ẑ*_5_ = 0.7071418. Compared the cascaded analysis model (38) with the complete model (39), the dynamic behavior of both models are quite similar as displayed in Figure 8, where the steady-state error between the two cases is less than 6%.

### Stability

The reference S-system for the signal transduction network is modeled as

(40) $$\;\begin{array}{l} {\dot{x}}_{1}=2x_{2}-1.2x_{1}^{0.5}x_{3}^{-1},\;\;\;x_{1}(0)=2\\ {\dot{x}}_{2}=2x_{1}^{0.1}x_{3}^{-1}x_{4}^{0.5}-2x_{2},\;\;\;x_{2}(0)=0.1\\ x_{3}=0.5\\ x_{4}=1 \end{array}$$

and the permissible ranges of the state variables are 1.3456 ≤ *x*_1_ ≤ 3.5861 and 0.1 ≤ *x*_2_ ≤ 2.2724.

Performing the second-order Taylor expansion for the S-system (40) around the steady state gives the linearized S-system:

(41) $$\begin{array}{c} {\dot{x}}_{1}=-0.9505x_{1}+2x_{2}+7.3853+0.0442x_{1}^{2},\\ {\dot{x}}_{2}=-2x_{1}-0.3631x_{2}+7.8019-0.0756x_{1}^{2} \end{array}$$

Consider (41). in the compact matrix form as

(42) $$\begin{array}{l} \;\left[\begin{array}{c} {\dot{x}}_{1}\\ {\dot{x}}_{2} \end{array}\right]=\left[\begin{array}{cc} -0.9505 & 2\\ -2 & -0.3631 \end{array}\right]\left[\begin{array}{c} x_{1}\\ x_{2} \end{array}\right]\\ \;\;\;\;\;\;\;\;\;\;\;\;\;\;\;\;\;\;\;+\left[\begin{array}{c} 7.3853\\ 7.8019 \end{array}\right]+\left[\begin{array}{c} 0.0442x_{1}^{2}\\ -0.0378x_{1}^{2} \end{array}\right] \end{array}$$

where the system eigenvalues are −0.6052 and −2.3453 and the residual errors satisfy

(43) $$\begin{array}{c} \;\left\|\left[\begin{array}{c} 0.0442x_{1}^{2}\\ -0.0378x_{1}^{2} \end{array}\right]\right\|\leq \alpha {\left\|\left[\begin{array}{c} x_{1}\\ x_{2} \end{array}\right]\right\|}_{2}\\ \Rightarrow 0.0038x_{1}^{4}\leq \alpha^{2}(x_{1}^{2}+x_{2}^{2}\;) \end{array}$$

For the extreme case, one can find the permissible lower bound of *α* is 0.0827.

To discuss the robust stability of (42), we choose *Q* = *I*_2_. According to the Lyapunov stability theory, there is a unique *P* = *P^T^* > 0 to (16) as

(44) $$P=\left[\begin{array}{cc} 1.4852 & -0.2058\\ -0.2058 & 1.6204 \end{array}\right]$$

From (17), the permissible range of *α* ensuring stability is given by

(45) $$\alpha <\frac{\lambda_{\text{min}}(Q)}{\lambda_{\text{max}}(P)}=0.6588$$

That is, the system would remain its stability provided that 0.0827 ≤ *α* ≤ 0.6588.

### Control design

**Figure 9.**
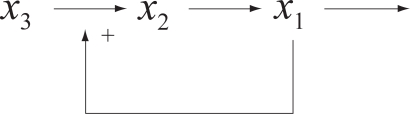
Reference system for the control design.

Consider the signal transduction network illustrated in Figure 9. Let one of the independent variable *x*_3_ be the control variable and the dependent variables *x*_1_ and *x*_2_ be the state variables. In order to discern the control variable and state variables, we define the control variable as *u*. Then the S-system can be written as follows

(46) $$\begin{array}{l} {\dot{x}}_{1}=2x_{2}-2.4x_{1}^{0.5},\\ {\dot{x}}_{2}=4x_{1}^{0.1}x_{3}-2x_{2},\\ x_{3}=u \end{array}$$

Now one can compute the linearization as the following steps. First, the system can be expressed in the control format as

(47) $$\dot{x}=f(x)+l(x)u$$

where

(48) $$f(x)=\left[\begin{array}{c} 2x_{2}-2.4x_{1}^{0.5}\\ -2x_{2} \end{array}\right],\;\;\;l(x)=\left[\begin{array}{c} 0\\ 4x_{1}^{0.1} \end{array}\right]$$

For the nominal system (47), a coordinate transformation exists as

(49) $$\left[\begin{array}{c} z_{1}\\ z_{2} \end{array}\right]=\left[\begin{array}{c} \psi_{1}(x)\\ \psi_{2}(x) \end{array}\right]=\left[\begin{array}{c} h(x)\\ L_{f}h(x) \end{array}\right]$$

where *h*(*x*) = *x*_1_, which satisfies $\frac{\partial h}{\partial x}l=0$, $\frac{\partial (L_{f}h)}{\partial x}l\neq 0$. That is, setting

(50) $$\left[\begin{array}{c} z_{1}\\ z_{2} \end{array}\right]=\left[\begin{array}{c} x_{1}\\ 2x_{2}-2.4x_{1}^{0.5} \end{array}\right]$$

transforms the original state-space representation into

(51) $$\left[\begin{array}{c} {\dot{z}}_{1}\\ {\dot{z}}_{2} \end{array}\right]=\left[\begin{array}{cc} 0 & 1\\ 0 & 0 \end{array}\right]\left[\begin{array}{c} z_{1}\\ z_{2} \end{array}\right]+\left[\begin{array}{c} 0\\ 1 \end{array}\right]v$$

Next, select the new control input

(52) $$v=-[\begin{array}{cc} k_{1} & k_{2} \end{array}]z$$

and substitute this into (51) to give

(53) $$\dot{z}=A_{c}z$$

where

(54) $$A_{c}=\left[\begin{array}{cc} 0 & 1\\ -k_{1} & -k_{2} \end{array}\right]$$

On the basis of (51), the control law for the original can be chosen as

(55) $$u=\frac{v-L_{f}^{2}h(x)}{L_{l}L_{f}h(x)}$$

The S-system with control can then be expressed in the closed-loop configuration as

(56) $$\begin{array}{l} {\dot{x}}_{1}=2x_{2}-2.4x_{1}^{0.5},\\ {\dot{x}}_{2}=4x_{1}^{0.1}u-2x_{2},\\ u\\ =\frac{-k_{1}x_{1}-k_{2}(2x_{2}-2.4x_{1}^{0.5})+2.4x_{1}^{0.5}x_{2}+4x-2.88}{8x_{1}^{0.1}} \end{array}$$

**Figure 10.**
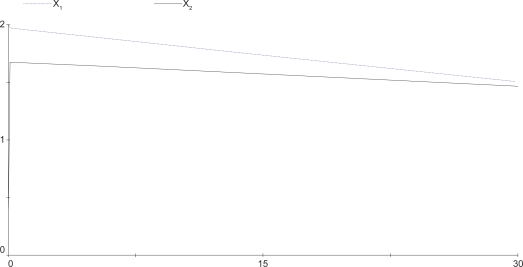
Dynamic simulation for the case of K = [1 100].

Letting *K* = [1 100] yields the transient responses illustrated as in Figure 10. The result shows the effect of control input for signal transduction.

Next, consider the system (47) with uncertainties and Δf^*^ = 0.1. On the basis of (25)–(30) and (48)–(54), one can derive a linearizd system as follows

(57) $$\dot{z}=\left[\begin{array}{cc} 0 & 1\\ -k_{1} & -k_{2} \end{array}\right]z+\left[\begin{array}{c} 0\\ 1 \end{array}\right]\Delta B(z)$$

where Δ*B*(*z*) = (8*x*_1_^0.1^ Δf*) ∘ ψ^−1^ (*z*). Select *K* = [10 100] then (57) becomes

(58) $$\dot{z}=\left[\begin{array}{cc} 0 & 1\\ -10 & -100 \end{array}\right]z+\left[\begin{array}{c} 0\\ 0.8z_{1}^{0.1} \end{array}\right]$$

We now choose *Q* = *I_2_* and solve *P* from (33) as follows

(59) $$P=\left[\begin{array}{cc} 5.0550 & 0.0500\\ 0.0500 & 0.0055 \end{array}\right]$$

From (35), we have

(60) $$\frac{0.08z_{1}^{1.1}+0.0088z_{1}^{0.1}z_{2}}{z_{1}^{2}+z_{2}^{2}}\leq \alpha_{1}$$

and from (36), we have

(61) $$\alpha <\lambda_{\text{min}}(Q)\Rightarrow \alpha_{1}<1$$

That is, if *α*_1_ satisfies (60) and (61), then the system would be robustly stable.

Second, we choose *Q* = 0.5*I*_2_ and solve *P* as

(62) $$P=\left[\begin{array}{cc} 2.52750 & 0.02500\\ 0.02500 & 0.00275 \end{array}\right]$$

From (35), we have

(63) $$\frac{0.04z_{1}^{1.1}+0.0044z_{1}^{0.1}z_{2}}{z_{1}^{2}+z_{2}^{2}}\leq \alpha_{2}$$

and from (36), we have

(64) $$\alpha <\lambda_{\text{min}}(Q)\Rightarrow \alpha_{2}<0.5$$

We examine the value of *Q* to the permissible range of *α*. Let the permissible ranges of *z*_1_ and *z*_2_ are 0.0604 ≤ *z*_1_ ≤ 0.9141 and −0.0836 ≤ *z*_2_ ≤ 0, respectively. Considering the extreme case of (60), one can find

(65) $$0.0852\leq \alpha_{1}$$

According to (61) and (65), the permissible range of *α*_1_ is then given by

(66) $$0.0852\leq \alpha_{1}<1$$

Similarly, the permissible range of *α*_2_ is

(67) $$0.0426\leq \alpha_{2}<0.5$$

On the basis of (66) and (67), one can easily find that the permissible range of *α* is proportional to the magnitude of *Q*.

### Control design for cascaded analysis model

**Figure 11.**
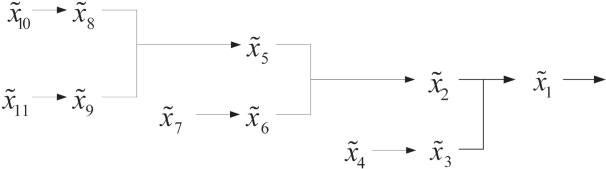
Reference S-system in control design for cascaded analysis model.

Consider the complete S-system as follows and the signal transduction pathways is shown in Figure 11.

$$\begin{array}{l} {\overset{\dot{˜}}{x}}_{9}=2{\tilde{x}}_{11}-2{\tilde{x}}_{8}^{1.01}{\tilde{x}}_{9},\\ {\overset{\dot{˜}}{x}}_{8}=2{\tilde{x}}_{10}-2{\tilde{x}}_{8}^{0.5}{\tilde{x}}_{9}^{0.5},\\ {\overset{\dot{˜}}{x}}_{6}=2{\tilde{x}}_{7}-2{\tilde{x}}_{8}^{1.01}{\tilde{x}}_{9},\\ {\overset{\dot{˜}}{x}}_{5}=2{\tilde{x}}_{8}^{0.5}{\tilde{x}}_{9}^{0.5}-2{\tilde{x}}_{5}^{0.5}{\tilde{x}}_{6}^{0.5},\\ {\overset{\dot{˜}}{x}}_{3}=2{\tilde{x}}_{4}-2{\tilde{x}}_{2}^{1.01}{\tilde{x}}_{3},\\ {\dot{\tilde{x}}}_{2}=2{\tilde{x}}_{5}^{0.5}{\tilde{x}}_{6}^{0.5}-2{\tilde{x}}_{2}^{0.5}{\tilde{x}}_{3}^{0.5},\\ {\overset{\dot{˜}}{x}}_{1}=2{\tilde{x}}_{2}^{0.5}{\tilde{x}}_{3}^{0.5}-2{\tilde{x}}_{1},\\ {\tilde{x}}_{4}={\tilde{x}}_{7}={\tilde{x}}_{10}={\tilde{x}}_{11}=1 \end{array}$$

**Figure 12.**
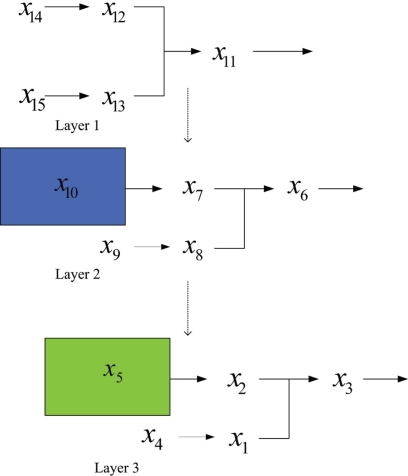
Three-layered cascaded analysis model.

The S-system can be further expressed in a three-layered cascaded analysis model shown as in Figure 12. We construct the first layered S-system as follows

$$\begin{array}{l} {\dot{x}}_{13}=2x_{15}-2x_{1}^{1.01}x_{2},\\ {\dot{x}}_{12}=0.5x_{14}-0.5x_{12}^{0.5}x_{13}^{0.5},\\ {\dot{x}}_{11}=2x_{12}^{0.5}x_{13}^{0.5}-2x_{11} \end{array}$$

The output concentration of the S-systems is *x*_11_ = 1. On the basis of the output concentration, we proceed to construct the second layer as

$$\;\;\begin{array}{c} {\dot{x}}_{8}=2x_{9}-2x_{7}^{1.01}x_{8},\\ {\dot{x}}_{7}=x_{10}-x_{7}^{0.5}x_{8}^{0.5},\\ {\dot{x}}_{6}=2x_{7}^{0.5}x_{8}^{0.5}-2x_{6} \end{array}$$

The output concentration of the S-systems is *x*_6_ = 1. On the basis of this model, we construct the third layer as

$$\begin{array}{l} {\dot{x}}_{1}=2x_{4}-2x_{1}^{1.01}x_{2},\\ {\dot{x}}_{2}=2x_{5}-x_{1}^{0.5}x_{2}^{0.5},\\ {\dot{x}}_{3}=2x_{1}^{0.5}x_{2}^{0.5}-2x_{3} \end{array}$$

Now select the independent variable *x*_4_ be the control variable, other independent variable be a constant and *x*_1_, *x*_2_, *x*_3_ be the state variables.^11^ We set all parameters then the S-system form can be written as follows

(68) $$\begin{array}{c} {\dot{x}}_{1}=u-2x_{1}^{1.01}x_{2},\\ x_{2}=1-2x_{1}^{0.5}x_{1}^{0.5},\\ {\dot{x}}_{3}=2x_{1}^{0.5}x_{1}^{0.5}-2x_{3} \end{array}$$

Now one can compute the linearized model. First, the system (68) is expressed in the control format as

(69) $$\dot{x}=f(x)+l(x)u$$

where

$$f(x)=\left[\begin{array}{c} -2x_{1}x_{2}\\ 1-2x_{1}^{0.5}x_{2}^{0.5}\\ 2x_{1}^{0.5}x_{2}^{0.5}-2x_{3} \end{array}\right],\;\;l(x)=\left[\begin{array}{c} 1\\ 0\\ 0 \end{array}\right]$$

For the nominal system (69), a coordinate transformation exists as follows

(70) $$\left[\begin{array}{c} z_{1}\\ z_{2}\\ z_{3} \end{array}\right]=\left[\begin{array}{c} \psi_{1}(x)\\ \psi_{2}(x)\\ \psi_{3}(x) \end{array}\right]=\left[\begin{array}{c} h(x)\\ L_{f}h(x)\\ L_{f}^{2}h(x) \end{array}\right]$$

where *h*(*x*) = *x*_2_ + *x*_3_, which stratifies $\frac{\partial h}{\partial x}l=0$, $\frac{\partial (L_{f}h)}{\partial x}l=0$, $\frac{\partial (L_{f}^{2}h)}{\partial x}l\neq 0$. That is, setting

(71) $$\left[\begin{array}{c} z_{1}\\ z_{2}\\ z_{3} \end{array}\right]=\left[\begin{array}{c} x_{2}+x_{3}\\ 1-2x_{3}\\ -4x_{1}^{0.5}x_{2}^{0.5}+4x_{3} \end{array}\right]$$

transforms the original state-space representation into

(72) $$\left[\begin{array}{c} {\dot{z}}_{1}\\ {\dot{z}}_{2}\\ {\dot{z}}_{3} \end{array}\right]=\left[\begin{array}{ccc} 0 & 1 & 0\\ 0 & 0 & 1\\ 0 & 0 & 0 \end{array}\right]\left[\begin{array}{c} z_{1}\\ z_{2}\\ z_{3} \end{array}\right]+\left[\begin{array}{c} 0\\ 0\\ 1 \end{array}\right]v$$

Next, selecting the new control input *v* = −*Kz* and substitute this into (72) gives

(73) $$\dot{z}=A_{c}z$$

where

$$A=\left[\begin{array}{ccc} 0 & 1 & 0\\ 0 & 0 & 1\\ -k_{1} & -k_{2} & -k_{3} \end{array}\right]$$

On the basis of (72) the control law for the original can be chosen as

(74) $$u=\frac{v-L_{f}^{3}h(x)}{L_{l}L_{f}^{2}h(x)}$$

The S-system with control can then be expressed in the closed-loop configuration as

(75) $$\begin{array}{l} {\dot{x}}_{1}=u-2x_{1}^{1.01}x_{2},\\ {\dot{x}}_{2}=1-2x_{1}^{0.5}x_{2}^{0.5},\\ {\dot{x}}_{3}=2x_{1}^{0.5}x_{2}^{0.5}-2x_{3},\\ u=\frac{-k_{1}(x_{2}+x_{3})-k_{2}(1-2x_{3})-k_{3}(-4x_{1}^{0.5}x_{2}^{0.5}+4x_{3})-4x_{1}^{0.5}x_{2}^{1.5}+2x_{1}^{0.5}x_{2}^{-0.5}-4x_{1}-8x_{1}^{0.5}x_{2}^{0.5}+8x_{3}}{-2x_{1}^{-0.5}x_{2}^{0.5}} \end{array}$$

**Figure 13.**
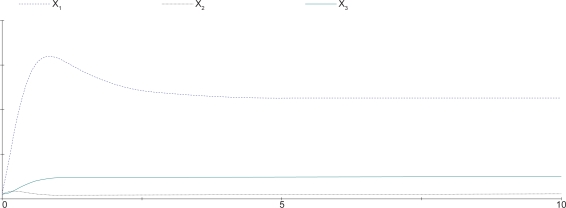
Dynamic simulation for the case of *K* = [1 10 40].

Letting *K* = [1 10 40] yields the transient responses illustrated as in Figure 13.

For the demonstrative purpose, consider the situation of Δf^*^ = 0.1. On the basis of (25)–(30) and (73), one can derive the linearized system as follows

(76) $$\dot{z}=\left[\begin{array}{ccc} 0 & 1 & 0\\ 0 & 0 & 1\\ -k_{1} & -k_{2} & -k_{3} \end{array}\right]z+\left[\begin{array}{c} 0\\ 0\\ 1 \end{array}\right]\Delta B(z)$$

where $\Delta B(z)=(-2x_{1}^{-0.5}x_{2}^{0.5}\Delta f^{*}){}^{\circ}\psi^{-1}(z)$. Select *K* = [1 10 20], then (76) becomes

$$\dot{z}=\left[\begin{array}{ccc} 0 & 1 & 0\\ 0 & 0 & 1\\ -1 & -10 & -20 \end{array}\right]z+\left[\begin{array}{c} 0\\ 0\\ 0.4\left(\frac{2z_{1}+z_{2}-1}{z_{3}+2z_{2}-2}\right) \end{array}\right]$$

We choose *Q* = *I*_3_ and compute *P* from solving (33)

$$P=\left[\begin{array}{ccc} 6.0578 & 10.0779 & 0.5000\\ 10.0779 & 21.4347 & 1.0578\\ 0.5000 & 1.0578 & 0.0779 \end{array}\right]$$

From (35), we have

$$\frac{2\left(0.2z_{1}\left(\frac{2z_{1}+z_{2}-1}{z_{3}+2z_{2}-2}\right)\right)+0.42312z_{2}\left(\frac{2z_{1}+z_{2}-1}{z_{3}+2z_{2}-2}\right)+0.03116z_{3}\left(\frac{2z_{1}+z_{2}-1}{z_{3}+2z_{2}-2}\right)}{z_{1}^{2}+z_{2}^{2}+z_{3}^{2}}\leq \alpha$$

as and from (36), we have

$$\alpha <\lambda_{\text{min}}(Q)\Rightarrow \alpha \leq 1$$

That means the system under control would be robustly stable.

## Conclusion

This paper proposes a method for constructing the dynamic model of signal transduction networks and a primary control design has been proposed for the system. A cascaded analysis model for constructing the signal transduction network model has been proposed. The advantage of cascaded analysis model is that the model preserved the major dynamic feature of the S-systems with a simplified model while avoiding much computation burden. The stability condition for the linearized S-system has been derived. A method for controlling the steady state to the desired level using the technique of feedback linearization has also been attempted. The development of this issue is undergoing theoretical investigation and experimental verification.
